# Comparison of Caffeine and d-amphetamine in Cocaine-Dependent Subjects: Differential Outcomes on Subjective and Cardiovascular Effects, Reward Learning, and Salivary Paraxanthine

**DOI:** 10.4172/2155-6105.1000176

**Published:** 2014-03-06

**Authors:** Scott D Lane, Charles E Green, Joy M Schmitz, Nuvan Rathnayaka, Wendy B Fang, Sergi Ferré, F Gerard Moeller

**Affiliations:** 1 Center for Neurobehavioral Research on Addictions, Department of Psychiatry & Behavioral Sciences, University of Texas Health Science Center – Houston, Houston, TX USA; 2 Center for Human Toxicology, University of Utah, Salt Lake City, UT, USA; 3 Integrative Neurobiology, Intramural Research Program, National Institute on Drug Abuse, Baltimore, MD, USA; 4 Division on Addictions, Department of Psychiatry, Virginia Commonwealth University, Richmond, VA, USA

**Keywords:** Caffeine, d-amphetamine, Paraxanthine, Cocaine dependence, Human, Subjective effects, Cardiovascular effects, Reward learning, Adenosine A1/A2 receptor

## Abstract

Due to indirect modulation of dopamine transmission, adenosine receptor antagonists may be useful in either treating cocaine use or improving disrupted cognitive-behavioral functions associated with chronic cocaine use. To compare and contrast the stimulant effects of adenosine antagonism to direct dopamine stimulation, we administered 150 mg and 300 mg caffeine, 20 mg amphetamine, and placebo to cocaine-dependent vs. healthy control subjects, matched on moderate caffeine use. Data were obtained on measures of cardiovascular effects, subjective drug effects (ARCI, VAS, DEQ), and a probabilistic reward-learning task sensitive to dopamine modulation. Levels of salivary caffeine and the primary caffeine metabolite paraxanthine were obtained on placebo and caffeine dosing days. Cardiovascular results revealed main effects of dose for diastolic blood pressure and heart rate; follow up tests showed that controls were most sensitive to 300 mg caffeine and 20 mg amphetamine; cocaine-dependent subjects were sensitive only to 300 mg caffeine. Subjective effects results revealed dose × time and dose × group interactions on the ARCI A, ARCI LSD, and VAS ‘elated’ scales; follow up tests did not show systematic differences between groups with regard to caffeine or d-amphetamine. Large between-group differences in salivary paraxanthine (but not salivary caffeine) levels were obtained under both caffeine doses. The cocaine-dependent group expressed significantly higher paraxanthine levels than controls under 150 mg and 3–4 fold greater levels under 300 mg at 90 min and 150 min post caffeine dose. However, these differences also covaried with cigarette smoking status (not balanced between groups), and nicotine smoking is known to alter caffeine/paraxanthine metabolism via cytochrome P450 enzymes. These preliminary data raise the possibility that adenosine antagonists may affect cocaine-dependent and non-dependent subjects differently. In conjunction with previous preclinical and human studies, the data suggest that adenosine modulating drugs may have value in the treatment of stimulant use disorders.

## Introduction

Chronic cocaine use results in measurable disruptions in the (DA) dopamine system. Allostatic shifts in neural and behavioral systems result in neurobehavioral states that foster continued use and relapse [1]. Accordingly, compounds that directly modulate DA have been considered in cocaine dependence with the aim of regulating DA neurotransmission [2,3], with some laboratory and clinical evidence for efficacy [4–6]. It is presently unknown whether compounds that indirectly modulate dopamine will yield efficacy with regard to either reductions in cocaine use, or improvement in cognitive/behavioral processes that are disrupted following chronic cocaine use [7]. Because cocaine may be cross-tolerant with other direct dopamine modulators [8,9], and comprehensive reviews indicate mixed evidence for efficacy [10] indirect modulators should warrant consideration. Within-subject comparison of the psychopharmacological and cardiovascular effects of both direct and indirect modulators has not been extensive [6,11]. Subsequently, the manner in which these agents modulate the behavior of stimulant-dependent individuals and healthy non-addicted controls has not been broadly characterized.

Caffeine is a mixed A_1_ and A_2_ receptor antagonist that appears to modulate dopamine function at both presynaptic and postsynaptic sites [12]. Adenosine A_1_ and A_2_ antagonists have been suggested as potential pharmacotherapies in both substance abuse [13,14] and Parkinson’s disease [15,16]. The A_2A_ receptor antagonist SYN115 produced modest stimulant-like subjective effects [17] and enhanced fMRI-measured BOLD response in the orbital frontal cortex [2]. Ferré et al. have reported that both A_1_ receptor antagonists and paraxanthine (the primary metabolite of caffeine) have unique neurobiological and behavioral interactions with DA transmission [18,19].

Because adenosine modulation of DA is indirect, and because cocaine-dependence alters DA transmission and reward processing, adenosine antagonists may reveal unique psychopharmacological effects compared to direct DA modulators in cocaine-dependent subjects relative to non-stimulant using controls. In non-cocaine dependent subjects trained to discriminate cocaine, oral caffeine produced discrimination of cocaine-like effects [20]; however, cocaine was administered in oral preparation and at low doses. It is not clear how well these data generalize to caffeine effects in individuals who abuse cocaine. In individuals with past cocaine dependence, repeated within-day caffeine administration did not produce cocaine-like cardiovascular effects, but did produce higher stimulant-like subjective effects in the past-cocaine group relative to controls [21]. In subjects with a history of cocaine and nicotine use receiving IV nicotine and IV caffeine, [22] observed comparatively less robust effects of caffeine relative to nicotine. Rush et al., [23] found stimulant-like subjective effects, e.g., increases in rating of “liking” and “high”, following administration of IV caffeine in individuals with a history of stimulant abuse. Data from preclinical models are consistent with prior human data, DA D_1_ and D_2_ agonists partially or fully substituted for low-dose (10 mg/kg) caffeine in rats trained to discriminate caffeine from saline, while D_1_ and D_2_ antagonists did not generalize to caffeine [24]. The authors concluded that the discriminative effects of caffeine are at least partly dependent on DA receptors. This conclusion is supported in part by results showing that caffeine administration reinstates previously extinguished cocaine self-administration [25].

On the other hand, while the mechanism of action of cocaine (DA reuptake blocker) and d-amphetamine (DA release) are not identical, both act directly on DA systems. In stimulant abusers, d-amphetamine administration results in stimulant-like subjective effects in dose-dependent fashion [26]. In highly controlled, rigorous testing protocols using a range of acute doses, d-amphetamine and methamphetamine effects are generally robust in stimulant abusers [27,28]. However, in heavy cocaine abusers who smoke crack cocaine at least twice per week, the effects of acute d-amphetamine were blunted [29].

In cocaine-dependent subjects, to our knowledge adenosine effects have not been directly compared to stimulants like d-amphetamine that act directly on the DA system. The question may be of interest because both DA agonists and, more recently, adenosine antagonists have been considered in the treatment of cocaine dependence. To this end, the present study examined the subjective and effects of both caffeine and d-amphetamine in cocaine dependent individuals and healthy controls. We also examined reward learning using a task developed by Frank and colleagues, with demonstrated sensitivity to dopamine function, including Parkinson’s disease and administration of cabergolide, haloperidol, and amphetamine [30–33].

Based on preclinical data suggesting unique behavioral and neurobiological (A_1_/A_2A_) properties of paraxanthine and caffeine [12,19], salivary caffeine and paraxanthine levels were examined at multiple time points following placebo and caffeine dosing. The examination of salivary caffeine and paraxanthine was exploratory in nature, and undertaken to provide additional information about possible differential effects of A_1_ and A_2_. Because caffeine is a mixed A_1_/A_2_ antagonist but the primary metabolite paraxanthine is a specific A_2A_ antagonist [19], expression of saliva levels between groups could shed light on unique between-group profiles of cardiovascular and subjective effects to caffeine.

Because of documented dopamine dysfunction and expected tolerance to the effects of d-amphetamine in subjects with chronic cocaine exposure [3,20], we anticipated that the cocaine group would show less sensitivity in subjective and cardiovascular effects following d-amphetamine administration relative to caffeine. Conversely, based on the limited stimulant use history and moderate caffeine use history in a control group, we expected that controls would show greater subjective and cardiovascular sensitivity to the effects of d-amphetamine relative

## Materials and Methods

This study was approved by the Committee for the Protection of Human Subjects (the Institutional Review Board for the University of Texas Health Science Center at Houston).

### Subjects

Thirteen (3 female) non-treatment seeking cocaine dependent subjects and 10 (5 female) healthy control subjects were recruited using advertisements in local newspapers. After reading the consent document and providing written informed consent, all subjects underwent a screening consisting of a physical examination and complete blood count, urine pregnancy test (females), serum chemistries, and HIV test. All subjects also underwent a structured psychiatric interview using the Structured Clinical Interview for DSM-IV. Exclusion criteria for cocaine-dependent subjects included any current psychiatric disorder other than cocaine and nicotine dependence. Subjects were excluded for past dependence on all other substances except marijuana. Four cocaine-dependent subjects met criteria past cannabis use disorder. One cocaine dependent subject met criteria for past opiate abuse. Exclusion for controls included any current or past psychiatric disorder other than nicotine dependence. Non-psychiatric medical exclusion criteria included any clinically significant medical disorder that could disrupt laboratory performance or metabolic functioning, or a disorder requiring medication that could affect the central nervous system (e.g., hypertension, diabetes, and HIV). Subjects were excluded from participation if they had a positive breath alcohol screen or a positive urine drug screen for drugs of abuse other than cocaine on the day of the scan. Subjects were also excluded if they were pregnant. A psychiatrist screened all subjects for symptoms of cocaine intoxication if they had a positive urine drug screen for cocaine on the day of testing. No acute cocaine intoxication was observed. Inclusion criteria for cocaine dependent subjects included age 18–55 years, meeting current Diagnostic and Statistical Manual for Mental Disorders, Fourth Edition (DSM-IV) criteria for cocaine dependence, and not treatment seeking. All subjects had an ECG prior to testing/dosing, read by a cardiologist and approved for inclusion. All subjects had a BMI between 23–29 to minimize pharmacokinetic and pharmacodynamic variation. The groups were matched on moderate caffeine consumption in the form of coffee or caffeinated soda, and consumed a reported daily range of caffeine use between 50 mg and 250 mg, based on standard caffeine levels found in one soda and one cup of coffee; salivary caffeine levels did not vary between groups on placebo administration days (see results).

### Medication, dosing, and general design

Across five separate testing days, subjects were administered two placebo doses, 20 mg d-amphetamine, 150 mg caffeine, and 300 mg caffeine in counterbalanced fashion. The primary focus was on the effects of caffeine (related to adenosine and paraxanthine function) in cocaine dependent subjects. D-amphetamine was used as a positive comparison to caffeine. All doses were prepared in blue opaque #00 capsules and administered orally at 9:00 AM each morning of testing. Doses of d-amphetamine and caffeine were selected to be comparable based on previous laboratory studies of both drugs in stimulant abusers (as reviewed in the introduction).

The general design of the study was as follows. Subjects arrived at 8 AM and provided a urine sample and a self-report of recent drug use. At 8:30 and every 30 minutes after until 11:30 AM, blood pressure and heart rate values were obtained. At 8:30, 9:30, 10:30, and 11:30, saliva samples were obtained to measure salivary caffeine and paraxanthine. At 9:00 AM subjects were administered the capsule (placebo, caffeine, or d-amphetamine, as described above). At approximately 9:01 AM and 10:00, subjective effects measures were obtained. At 10:15 AM a probabilistic reward task was completed.

### Cardiovascular measures

Blood pressure and heart rate were obtained at seven time points, approximately every 30 minutes beginning at 8:30 AM and ending at 11:30 AM. A final heart rate and blood pressure reading, not included in the data analyses, was obtained just prior to release at approximately 12:30 PM. All measures were acquired via sphygmomanometer (BpTru Medical Devices, Coquitlam, CA).

### Subjective effects measures

The Addiction Research Center Inventory (ARCI), the Visual Analogue Scale (VAS) of drug effects, and a 5-item Drug Effects Questionnaire (DEQ) were used to assess subjective effects. The 49-item True/False ARCI short form was used [34]. It has been empirically derived to assess factors representing subjective drug effects divided into 5-scales: lysergic acid diethylamide (LSD) scale; amphetamine (A) scale; Benzedrine group (BG) scale; morphine–benzedrine group (MBG) scale; and the pentobarbital, chlorpromazine, alcohol group (PCAG) scale [35]. The Visual Analogue Scale of drug effects [36] asked subjects to rate adjectives regarding how they felt, including Anger, Anxiety, Depression, Vigor, Fatigue, Tension, Confusion, Elation, High, Nauseated, Hungry, and Stimulated. The Drug Effects Questionnaire (DEQ) [37,38] asked subjects to rate phrases related to subjective drug effects including, “I feel a good effect”, “I feel a bad effect”, “I like the like effect”, “I feel high”, and “I would like more.” For both the VAS and DEQ questions, an on-screen slider bar 100 mm in length was anchored by “not at all” on the left (0 mm), and “extremely” on the right (100 mm). All three subjective effects scales have well-established validity and sensitivity to stimulant drugs effects [39,40]. All subjective effects measures were acquired via automated computer programs in which the subject used the computer mouse to enter responses. The three measures were acquired at two points, once immediately after dosing (to serve as a baseline for that day) and once at approximately 60 minutes post-dosing.

### Probabilistic selection task

In the probabilistic feedback selection task [30,33], three different stimulus pairs (Japanese Hiragana characters, designated AB, CD, EF) were presented in random order. Subjects chose one of the two stimuli in the pair, and positive or negative feedback followed some choices. Feedback was probabilistic: on AB trials choosing stimulus A resulted in positive (“correct”) feedback on 80% of trials, and choosing B resulted in negative (“incorrect”) feedback on 80% of trials (vice versa for the remaining 20% of trials). In CD and EF pairs, choosing C resulted in positive feedback on 70% of trials, and choosing E resulted in positive feedback on 60% of EF trials. Over the course of training (or stage 1 acquisition), participants should learn to choose stimuli A, C, and E more often than B, D, or F. Learning to choose A vs. B could result from discriminating that A leads to positive feedback, that B leads to negative feedback, or both. To evaluate whether subjects learned more about positive or negative outcomes of their decisions, subjects were tested (stage 2) with novel combinations of stimulus pairs involving either an A (e.g., AC, AD, AE) or a B (e.g., BC, BD, BE) in the absence of feedback. Learning from positive or negative feedback should be associated with favoring stimulus A or avoiding the B stimulus, respectively, when presented with novel test pairs. Frank et al. [31,32] have shown that this task is sensitive to both tonic and phasic changes in dopamine levels. The task was given once at approximately 10:15 each test day, 75 minutes post-dose. Analysis of the stage 2 data requires that phase 1 data (acquisition) reach acceptable mastery criteria in order to make a valid interpretation of the stage 2 data (novel stimulus pairing tests). In the present dataset, more than half of all subjects in both groups failed to reach acceptable mastery criteria (better than chance). Therefore, data analysis focused on the phase 1 acquisition data to interpret effects on reward learning. Subjects were instructed that they would be paid based on the accuracy of their performance. All subjects were in fact paid an amount of money varying between $3.00 and $5.00 each test session, with the actual amount determined by a quasi-random computer generated value.

### Salivary caffeine and paraxanthine

Salivary caffeine and paraxanthine data were obtained at four time points (−30, +30, +90, and +150 minutes), and change over time was expected to be relevant to the between-group comparisons. Saliva samples were collected by having subjects chew and fully saturate oral fluid onto a swab collection device (Salimetrics^©^ oral swab, SOS, 2mL volume), centrifuged, and stored in individual collection tubes at −30 C. Frozen samples were sent to the Center for Human Toxicology Laboratory at the University of Utah, thawed and processed for quantitative analysis. Measurement was conducted utilizing sample preparation and high-performance liquid chromatography (HPLC) as described in previous human studies analyzing human saliva [41–43]. For the assay, the calibration curve range for the analyzed samples was 30 to 5000 ng/ml. Due to insufficient oral volume or breakage during sample transfer, data were lost or incomplete for two cocaine-dependent subjects. Analyses for the saliva data represent 11 cocaine-dependent subjects and 10 controls.

### Data analyses

Statistical analysis of the cardiovascular, subjective, and reward-learning data was first examined for violations of basic linear models assumptions (e.g., normality, heteroskedasticity, leverage) by inspection of the GLM test residuals via normal-quantile plots and Shapiro-Wilk tests. Violations were observed in all three datasets; data transformations did not resolve most violations. Thus, data for these variables were analyzed using general linear models with more conservative robust estimators (Huber-White sandwich), which are more resistant to distortions and provide better estimates under such conditions [44,45]. Residuals from the salivary caffeine and paraxanthine data analyses did not show violations of linear model assumptions; these data were analyzed using GLM with traditional standard error estimates.

Cardiovascular data were analyzed using linear models with the factors group (between), dose (repeated), and the group × dose interaction. For any significant dose, group, or dose × group/time interaction outcomes, appropriate pairwise contrasts and simple main effects were conducted using adjusted cell means with FDR correction for multiple comparisons. Because our initial hypotheses focused on differential relative effects of caffeine and d-amphetamine within each group, planned follow up within-group comparisons tests were utilized. Because cardiovascular measures routinely change across the day, and these data were obtained at seven time points, and we had no a priori assumptions regarding differential time × dose or time × dose × group interactions, for simplicity the reported cardiovascular effects did not include time as a variable.

Subjective effects data were analyzed using linear models with the factors group (between), dose (repeated), time (repeated), and the group × dose and group × dose × time interactions. Time was included in the subjective effects model because data were taken at two time points, pre-dosing and post-dose near peak expected effects (60–90 minutes), and thus we hypothesized differential change in subjective effects across groups pre- to post-dosing. Following significant main effects or interactions, planned follow up comparisons were conducted in the same manner as the cardiovascular data, in order to evaluate differential relative effects of caffeine and d-amphetamine within each group.

Probabilistic feedback selection task data were obtained only once per experimental test day. Accordingly, data were analyzed via GLM with the factors group (between), dose (repeated), and the group × dose interaction in the same manner as the cardiovascular data. Prior work utilizing this task to examine dopamine-relevant factors (e.g., clinical group, pharmacological challenge) has examined post-acquisition response proportions (e.g., “choose A” or “avoid B”) to interpret response patterns [30,31]. However, such analyses require that phase 1 data (acquisition) reach acceptable mastery criteria (better than chance) in order to make a valid interpretation of the stage 2 data (novel stimulus pairing tests). In the present dataset, more than half of all subjects, cocaine and control combined, failed to reach acceptable mastery criteria. Thus, we focused data analyses only on accuracy during the acquisition phase, in order to evaluate dose and group differences on acquisition in learning the stimulus pairs under probabilistic feedback. Accuracy was defined as selection of the stimulus associated with “correct” feedback on 80% of trials.

Saliva data were analyzed using linear models with the factors group (between), dose (repeated), time (repeated), and the group × dose and group × dose × time interactions. For significant main effects and interactions, appropriate pairwise contrasts and simple main effects were conducted using adjusted cell means with FDR correction for multiple comparisons. Additionally, if significant main or interaction effects were obtained, planned follow up comparisons tests were utilized to examine differences within each subject group, in order to evaluate differential relative effects of caffeine administration across the four time points in each day.

Because the groups were significantly different age, for all analyses age was initially included as a factor in the models. Age was not a significant factor in any of the GLM outcomes. For all statistical testing, effects size estimates are reported for significant main and interaction effects using the partial eta-squared $\left(\eta_{\rho }^{2}\right)$ method [46,47].

## Results

### Demographics

On average, cocaine dependent subjects were 43.38 (SD=7.88) years of age and controls were 33.5 (9.06), t (21)=2.79, p<.01. Cocaine dependent subjects completed an average of 12.35 (1.52) years of education and controls completed 13.45 (1.64), t (21)=−1.95, ns. Across both groups, none of the subjects were employed full time. Those employed part-time had working-class jobs (non-degree related). The cocaine group consisted of 10 African-American and three Caucasian individuals. The control group was comprised of eight African-Americans, one Caucasian, and one Hispanic. Ten of 13 cocaine-dependent subjects reported daily cigarette smoking, only three of 10 controls reported daily cigarette smoking, Fisher’s exact= .013. Among the 10 cocaine-dependent subjects who smoked, the average was 8.88 (7.15) cigarettes per day. Among the three controls, the average was 2.0 (1.64). The groups were not significantly different in gender representation, Fisher’s exact test=0.22. In the cocaine-dependent group, ASI drug history data were missing for three subjects. The average number of years of cocaine use for the remaining 10 subjects was 16.42 (range 13–22), and the average number of days using cocaine in the past 30 at study intake was 14.63 (range 4–20). Across test days, in the cocaine group 48 (74%) of urine samples were positive for benzoylecgonine, and 19 (29%) were positive for THC. One cocaine-group urine sample was positive for opiates. Self-reported last use of cocaine ranged from 11–36 hours from last laboratory visit. One control subject had a positive test for THC on one test day, a second control subject had positive test for opiates on one test day. Control subjects were negative for all other illicit substances. All subjects provided clean breath alcohol samples on all days of testing.

### Cardiovascular data

Table 1 provides results for the systolic BP, diastolic BP, and heart rate data. No significant main effects or interactions were obtained for systolic BP. For diastolic BP, no significant main effect of group or group × dose interaction was obtained, but there was a main effect of dose (F (3,22)=7.24, p<.002, $\eta_{\rho }^{2}=0.50$). Planned contrast tests within the control group revealed significant differences between placebo and 300 mg caffeine (*p* < .005, caffeine BP higher than placebo), placebo and 20 mg d-amphetamine (*p* < .05, d-amphetamine BP higher than placebo). Planned contrast tests within the cocaine group revealed significant differences between placebo and 300 mg caffeine (*p* < .005, caffeine BP higher than placebo), and between 300 mg caffeine and 20 mg d-amphetamine (*p* < .001, caffeine BP higher than d-amphetamine). Similar outcomes were obtained for heart rate data: no significant main effect of group or group × dose interaction, but a significant main effect of dose was obtained (F (3,22) = 8.11, *p* < .001, $\eta_{\rho }^{2}=0.56$). In the control group, planned follow-up contrast tests revealed significant differences between placebo and 150 mg (*p* < .001), 300 mg (*p* < .001), and 20 mg d-amphetamine (*p* < .05); all doses produced greater HR than placebo. Interestingly, no significant heart rate differences were found between any of the doses in the cocaine group. All dose effects remained significant after false discovery rate (FDR) correction for multiple comparisons.

### Subjective effects data

Table 2 summarizes data for the ARCI, DEQ, and VAS scales. Although the effects of group, dose and time were examined, in the interest of presenting a compendious table only the overall means of group and dose are presented in Table 2. In summary, few reliable or systematic subjective effects were observed. Only three subjective effects ratings revealed significant group × dose, dose × time, or group × dose × time interactions. There was a significant dose × time interaction on the ARCI A (amphetamine) scale (F (3, 22)=3.62, *p* <.03, $\eta_{\rho }^{2}=0.32$). Planned contrast tests showed a trend for differential change pre-dose (time 1) to post-dose (time 2) between the two groups under the placebo dose (*p* < .007, increased ratings for cocaine but not control) and the 300 mg caffeine dose (*p* < .02, increased ratings for cocaine but not control). After FDR correction for multiple comparisons, only the dose × time interaction for the ARCI A scale remained significant.

There were no significant interactions for the DEQ scales. For the VAS ‘elated’ scale there were significant interactions of dose × group (F (3, 22) = 3.16, p <. 05, η_ρ^2 = 0.30) and dose × time (F (3, 22) = 4.26, p < .02, η_ρ^2 = 0.37). Planned contrast tests showed between-group differences under the placebo (p < .04), 150 mg caffeine (p < .02), and 20 mg amphetamine (p = .05) doses, with cocaine users have higher ratings under each dose owing to higher baseline (pre-dose) ratings each day. More importantly, however, there were no differential relative effects within groups when examining change pre-dose (time 1) to post-dose (time 2) across doses.

### Probabilistic selection task data

Table 3 summarizes performance data (accuracy) in the acquisition stage for each group across the four doses. A significant group × dose interaction was not obtained, but there was a significant effect of dose (F (3, 22) = 3.15, p < .05). Planned contrast tests within each group revealed significant differences between doses. Within the control group, the 20 mg d-amphetamine dose produced greater accuracy than the other three doses: p < .02 vs. placebo, p < .03 vs. 150 mg caffeine, and p < .005 vs. 300 mg caffeine. Within the cocaine group, the 300 mg caffeine dose produced greater accuracy than placebo (p < .01).

### Salivary caffeine and paraxanthine data

**Figure 1:**
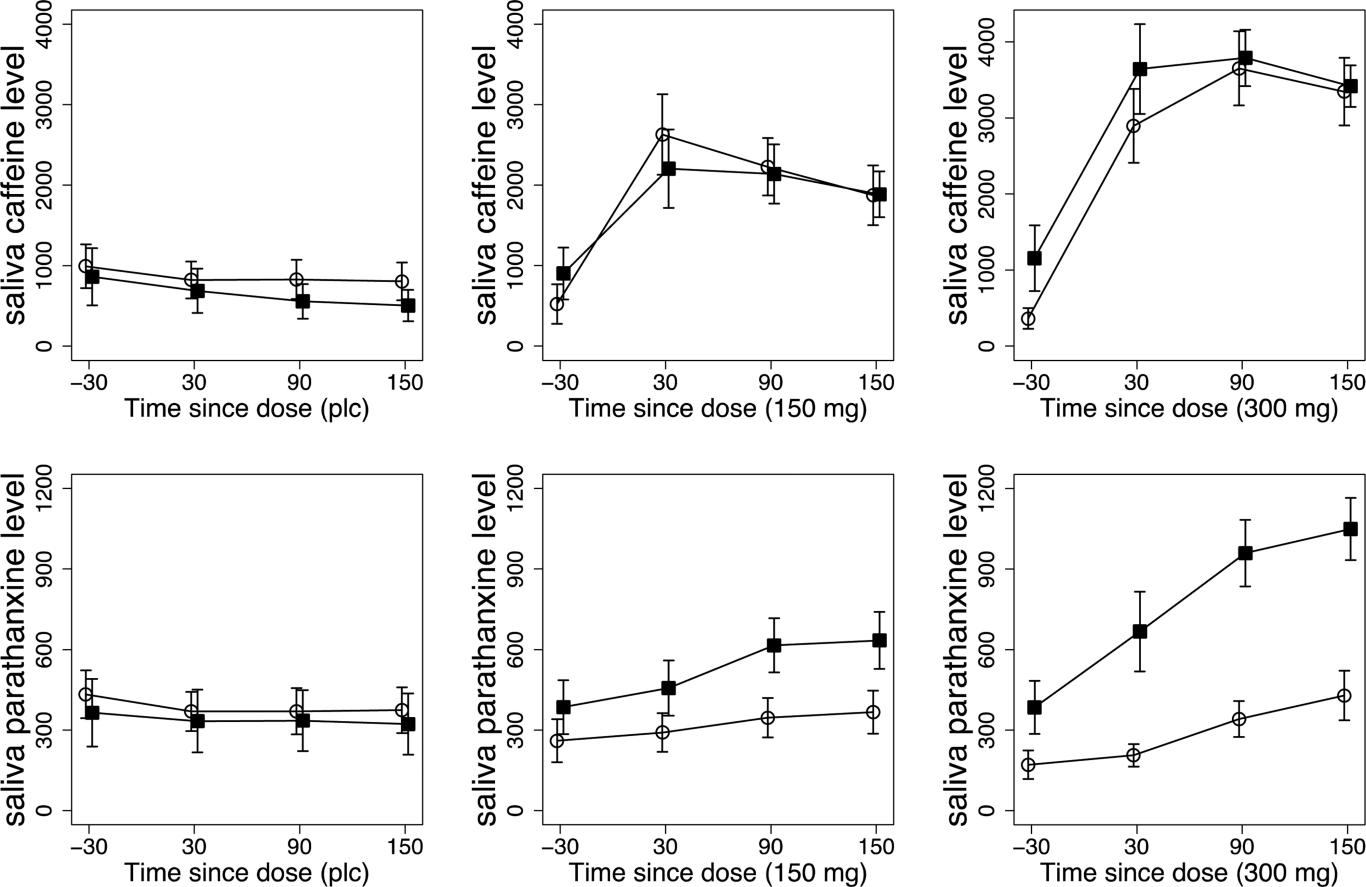
Salivary caffeine and paraxanthine levels acquired at −30, +30, +90, and +150 minutes post caffeine and placebo dosing for cocaine-dependent and control subjects. Time points are represented on the x-axis, and mean (± SEM) salivary levels (ng/mL) are shown on the y-axis.

Figure 1 presents comparisons of each group (N=11 cocaine, N=10 control) for caffeine and paraxanthine levels across four time points (−30 min pre-dose, and +30, +90, and +150 min post-dose) for the placebo, 150 mg and 300 mg caffeine doses. Clearly evident in Figure 1 is the lack of difference between groups in caffeine levels, and moderate (150 mg) to large (300 mg) differences between groups in paraxanthine levels. The cocaine group expressed substantially higher levels of salivary paraxanthine, which were dose-related and statistically significant. For salivary caffeine, there was a significant time × dose interaction (F (6, 126) = 29.42, p < .0001, $\eta_{\rho }^{2}=0.58$), but not a significant group × dose (F (2, 42) = 1.27) or group × time × dose interaction (F (6, 126) = 0.96). Follow-up tests revealed no relative differences within the groups on change in caffeine levels. For salivary paraxanthine, there was a significant group × dose × time interaction (F (6, 126) = 4.20, p < .004, $\eta_{\rho }^{2}=0.17$). Follow-up tests revealed no differences between groups at any time points under placebo, and significant differences between groups at +90 and +150 min under 150 mg caffeine, and +30, +90, and +150 mg under 300 mg caffeine (all p-values < .001); these remained significant after FDR correction.

To examine if any of the observe effects were related across the dependent measures, we constructed a maximum difference scores for each subject by calculating the difference between placebo vs. 300 mg caffeine 300 and placebo vs. 20 mg d-amphetamine based on the time point (in the case where multiple time points measures were obtained) where the maximum effects was observed. These values were obtained for each statistically significant dependent measure and entered into a canonical correlation analysis. No significant associations were obtained, suggesting the drug-related changes did no systematically co-vary across the measures.

## Discussion

Acute caffeine and d-amphetamine dosing produced a series of differential outcomes across the cocaine and control groups, but these outcomes were not systematic. Within the control group cardiovascular changes were observed between placebo and both 20 mg d-amphetamine and 300 mg caffeine, but in the cocaine group differences were only observed between placebo and 300 mg caffeine on the measure of diastolic BP. On the ARCI A scale, pre-dose to post-dose changes following d-amphetamine administration were observed only in the control group. The groups had different ratings on the VAS ‘elated’ scale following d-amphetamine and 150 mg caffeine administration. If any pattern is to be noted across the cardiovascular and subjective effects measures, it is that the cocaine group appeared more sensitive to the effects of 300 mg caffeine, while the control group appeared most sensitive to 20 mg d-amphetamine. Yet this conclusion should be interpreted as preliminary given the sample size and study limitations (see below).

For the control group, the results are largely consistent with previous reports of both d-amphetamine [48–50] and caffeine [51–53]. However, the subjective effects and cardiovascular outcomes for the cocaine-dependent subjects appear relatively blunted in comparison with previous studies of subjects with stimulant use disorders, both for caffeine [21,22,] and d-amphetamine effects [26,28,]. The present blunted effects of d-amphetamine are consistent with at least one recent report in users of smoked cocaine [29]. It is plausible to expect tolerance to the effects of d-amphetamine in cocaine dependent subjects relative to control subjects, as well as differential sensitivity to the effects of caffeine, owing to less extensive adaptation adenosine receptors relative to DA receptors. The cardiovascular effects of chronic cocaine use have been well documented, and include arrhythmias and other cardiovascular risks, some of which may be due to changes in cardiac tissue or compensatory processes leading to physiological adaptation and tolerance following repeated cocaine smoking [54,55].

Liguori et al. [1997] [22] found larger subjective effects of caffeine in subjects with past cocaine-dependence compared to controls, although they did not find differential effects of caffeine reinforcement between the groups. While Liguori et al. [1997] [22] suggested their results may have been due to sensitization to stimulants in the past cocaine dependent subjects, they did not use a positive control drug such a d-amphetamine. Additionally, direct comparison of the present results with Liguori et al., [1997] [22] is precluded by differences in drug use status (past vs. present cocaine dependence) and caffeine route of administration (hourly coffee administration vs. capsules at a single time point). Consistent with the present data, administration of IV caffeine to individuals with a history of stimulant abuse produced increases in positive mood and effects generally consistent with stimulant administration.

In stimulant abusing subjects, both methylphenidate and d-amphetamine produced dose-dependent stimulant-like effects [Stoops et al., 2004] [26]. We did not observe such robust outcomes; however, repeated dosing, greater dose ranging, more measurement time points, and more extensive training methodologies most likely produced better measurement sensitivity in Stoops et al. [26] than was achieved in the present study. Additionally, the drug use histories of the subject groups were not equivalent. Subjects in the present study had more extensive cocaine-use histories, which could have produced tolerance to the 20 mg dose of d-amphetamine. This point may be salient to the extent that caffeine, via activation of adenosine receptors, may produce a greater stimulant-like response than 20 mg d-amphetamine in more severe cocaine-dependent subjects, consistent with outcomes reported by Comer et al., [2013] [29]. It is likely that higher doses of d-amphetamine would have evidenced greater stimulant effects in the cocaine group [3]. Nonetheless, a unique feature of this study is the comparison of both direct (d-amphetamine) and indirect (caffeine) DA modulators and their relative effects within and between healthy control and cocaine-dependent subjects. To our knowledge, such comparisons have not been previously reported.

Previous work using the probabilistic feedback task reveals that the task is sensitive to manipulations via dopaminergic drugs [30,32]. For the control group, the enhanced accuracy rates are generally consistent with previous work showing performance-enhancing effects following d-amphetamine administration [33]. By contrast, in the cocaine dependent group 300 mg caffeine, but not d-amphetamine, improved task accuracy. Stimulant dependence is associated with anhedonia, disrupted reward processing, and decreased dopamine transmission in PFC [58], suggesting a mechanism by which the cocaine group did not show performance changes on the reward-based probabilistic feedback task following d-amphetamine administration. For example, Fillmore et al. [59] found that d-amphetamine dose-dependently worsened inhibitory control performance in stimulant abusers. Possibly, adenosine antagonism produced better cognitive enhancement than direct dopamine modulation. Unfortunately, direct comparisons with previous work using this same task and involving dopamine psychopharmacology and dopinergically-relevant disease groups is not entirely warranted. We necessarily focused data analyses on the acquisition stage rather than analyses of ‘choose A’ and ‘avoid B’ patterns drawn from novel test trial stage, which have been the target of computational and conceptual interpretations of dopamine modulation using this task [30].

Perhaps the most intriguing and consistent trends in the data were the differences in expression of paraxanthine and caffeine levels between the groups. While no differences in caffeine levels were observed between the groups, the cocaine-dependent group showed much higher levels of salivary paraxanthine. These differences in paraxanthine levels were dose related in both magnitude and time course (Figure 1), and may provide a plausible mechanism for the differential response to caffeine between the groups. At time points where salivary caffeine levels were equivalent (90 and 150 minutes post-dose), paraxanthine levels were notably higher in cocaine-dependent subjects compared to controls. Adenosine A2A receptors form functional heteromers with dopamine D2 receptors located in the striatum, altering the binding and signaling properties of D2 receptors [12,19]. It may therefore be inviting to speculate that striatal D2 neuroplasticity resulting from chronic cocaine use produces corresponding adaptations in adenosine receptor expression, occasioning the differential paraxanthine levels between groups. In vitro and preclinical rodent studies indicate that A2 receptor expression is altered by exposure to cocaine (including self-administration), particularly in the nucleus accumbens and dorsal striatum [59,60]. However, no studies of which we are aware have documented differential A1/A2 expression in cocaine-dependent human subjects related to acute or chronic cocaine exposure. Thus while plausible, this idea is currently untested and cannot be adequately addressed in the present dataset.

One major limitation in this study was that nicotine smoking was confounded with group status (Table 1, Section 3.1), where the cocaine group included more and heavier smokers than the control group. Regular nicotine use elevates the production of the cytochrome P450 enzyme CYP1A2, which in turn facilitates the metabolism of many drugs including caffeine [61]. Thus, the differential paraxanthine levels observed between the groups could have been mediated in large part by nicotine use, leaving the influence of smoking on paraxanthine levels impossible to separate from cocaine-use status. It is also well established that caffeine alters P450 CYP1A2 expression [62], and thus could have influenced the results. However, the groups were matched on moderate caffeine use, and thus this variable should not have influenced the present results. Rank-order correlations between nicotine use rates and the primary dependent measures (including paraxanthine levels) did not reveal any significant relationships; such relationships are necessary conditions for the presence of confounding variables, e.g., nicotine use [63]. However, correlational analyses are generally inadequate with small sample sizes, and the absence of significant relationships does not allay this ambiguity. From a therapeutic standpoint the group difference may not be relevant; the majority of individuals with cocaine dependence use nicotine regularly [64]. However, the differential smoking status between the groups hindered the experimental rigor and scientific coherence of the results, particularly for the paraxanthine outcomes. An improved design in future work will feature a second control group matched to the cocaine group on nicotine use but without other illicit substance abuse.

## Limitations

In addition to control for nicotine use rates between the groups, several other limitations must be considered. All subjects were moderate caffeine users to facilitate matching of groups, but inclusion of a broader range of caffeine use, from those who are minimal users to those who are caffeine dependent would arguably render more informative data with regards to differences between cocaine-dependent and control subjects, and variation in the effects of adenosine in cocaine dependence. The relatively small sample size likely contributed to variability in outcomes across measures. Our power to detect significant effects was clearly limited with sample sizes of 13 and 10. Another limitation of the sample size was the inability to examine gender differences, as gender and menstrual cycle phase are known to modulate the acute effects of d-amphetamine [65,66]. Having only 8 female subjects precluded the ability to test for gender effects, and failing to control for potential gender effects likely contributed unwanted variability and weakened the ability to detect important outcomes. Several variables were measured at only one or two time points. We intended to measure caffeine and d-amphetamine effects within their window of peak effect [21,67–69]. However, peak effects can vary across individuals and drugs, with caffeine reaching peak plasma levels in humans between 15–120 min, with a T1/2 = 2.5 – 4.5 hrs [70] and amphetamine reaching peak levels between 40–210 minutes, with a T1/2 = 11–13 hrs [71]. While we did observe differences between groups and across drugs, the finding were limited, and testing across a broader range of time points may well have revealed more systematic outcomes. Finally, peak paraxanthine levels were observed at 150 minutes post dosing and all subjective and performance data were obtained between 60 and 90 minutes. Therefore, to the extent that paraxanthine levels mediated the effects of caffeine administration (at least in cocaine-nicotine users), a more informative series of outcomes may have been obtained if the testing window had been extended beyond 90 minutes.

## Summary

We anticipated differential change between the groups when comparing d-amphetamine and caffeine, due to changes in dopamine function that accompany chronic cocaine use and possible cross-tolerance between cocaine and d-amphetamine [8]. However, the idiosyncratic patterns of change across measures, doses, and groups limit interpretation of the observed patterns of change. Despite inconsistent outcomes and current study limitations, there appears potential utility of adenosine modulating drugs in cocaine pharmacotherapy. It is hoped that the current data in union with previous investigation of the specific A2A antagonist SYN115 and parathanxine [17,2,14], will provide continued interest in the psychopharmacological investigation of adenosine modulators in cocaine dependence. To the extent that modulation of dopamine has shown promise as a pharmacotherapy for cocaine dependence [5,72,6,4], the use of A2A antagonists may be efficacious either as adjunctive supplements to direct dopamine modulators, or as monotherapies in patients for whom direct modulators are not indicated medically or are ineffective.

## Figures and Tables

**Table 1:** Effects sizes are provided for statistically significant outcomes.

Variable	Group	Dose				
		placebo	caffeine 150	caffeine 300	d-amph 20	**effect size** $\eta_{\rho }^{2}$
systolic BP	control	115.64	115.90	117.36	119.27	
		(3.41)	(4.07)	(4.20)	(2.13)	
	cocaine	120.18	122.10	123.84	120.30	
		(1.40)	(1.81)	(2.18)	(1.58)	
diastolic BP *	control	74.82	75.33	77.94	77.83	0.49
		(2.78)	(2.72)	(1.42)	(1.45)	
	cocaine	79.66	79.98	82.81	78.16	
		(3.01)	(1.79)	(1.74)	(1.56)	
heart rate **	control	66.10	62.47	61.33	65.55	0.53
		(1.88)	(2.09)	(1.67)	(1.23)	
	cocaine	63.08	62.60	61.57	63.51	
		(2.11)	(3.00)	(1.40)	(2.99)	

Means (delta-method robust standard errors) for cardiovascular measures across placebo, 150 mg caffeine, 300 mg caffeine, and 20 mg d-amphetamine doses. Data are averaged across the seven time points on each dose day, and shown separately for control and cocaine-dependent groups

* *p* < .05, main effect of dose, see text for details

** *p* < .001, main effect of dose, see text for details

**Table 2:** Effects sizes are provided for statistically significant outcomes

Variable	Group	Dose				
		placebo	caffeine 150	caffeine 300	d-amph 20	**effect size** $\eta_{\rho }^{2}$
ARCI-PCAG	control	3.87	3.62	3.52	3.64	
		(0.82)	(0.83)	(0.79)	(0.71)	
	cocaine	1.95	1.83	2.95	2.43	
		(0.58)	(0.68)	(0.82)	(0.67)	
ARCI-A *	control	3.20	3.60	3.80	3.22	0.33
		(0.51)	(0.68)	(0.59)	(0.38)	
	cocaine	3.30	3.39	3.19	3.69	
		(0.51)	(0.54)	(0.44)	(0.45)	
ARCI-BG	control	6.00	6.35	6.05	5.83	
		(0.39)	(0.56)	(0.57)	(0.36)	
	cocaine	5.69	5.77	5.07	5.57	
		(0.34)	(0.37)	(0.61)	(0.51)	
ARCI-MGB	control	4.09	4.34	5.14	4.04	
		(1.26)	(1.31)	(1.37)	(1.28)	
	cocaine	5.29	5.62	4.31	5.18	
		(1.13)	(1.23)	(0.96)	(1.00)	
ARCI-LSD ***	control	1.78	2.43	1.23	1.70	0.32
		(0.44)	(0.56)	(0.41)	(0.41)	
	cocaine	0.89	0.48	1.05	0.98	
		(0.47)	(0.49)	(0.49)	(0.45)	
VAS-depression	control	0.10	0.10	0.41	0.10	
		(0.10)	(0.10)	(0.30)	(0.06)	
	cocaine	1.05	0.62	1.96	1.10	
		(0.54)	(0.47)	(1.12)	(0.51)	
VAS-fatigue	control	3.85	3.00	3.44	3.60	
		(1.37)	(1.48)	(1.64)	(1.07)	
	cocaine	0.68	0.54	1.69	1.17	
		(0.32)	(0.25)	(0.71)	(0.36)	
VAS-tension	control	1.05	1.60	1.62	1.53	
		(0.38)	(0.53)	(0.60)	(0.48)	
	cocaine	1.63	0.96	2.38	1.88	
		(0.62)	(0.57)	(0.94)	(0.61)	
VAS-vigor	control	8.25	8.50	9.78	8.28	
		(2.06)	(1.92)	(1.98)	(2.01)	
	cocaine	10.42	10.85	9.27	10.31	
		(1.54)	(1.59)	(1.60)	(1.33)	
VAS-anxious	control	10.15	10.45	8.50	11.68	
		(5.31)	(5.69)	(4.91)	(4.62)	
	cocaine	19.64	21.35	25.27	21.71	
		(6.17)	(6.01)	(7.12)	(5.43)	
VAS-elated *, **	control	8.65	9.85	13.65	10.43	0.30, 0.37
		(5.33)	(5.32)	(6.10)	(4.48)	
	cocaine	25.99	32.54	21.65	25.71	
		(6.01)	(7.62)	(5.68)	(6.42)	
DEQ- feel effects	control	16.75	20.25	9.45	11.37	
		(7.58)	(6.31)	(4.97)	(4.93)	
	cocaine	17.30	20.73	17.62	18.68	
		(5.80)	(5.95)	(5.91)	(6.08)	
DEQ- like effects	control	39.92	35.62	52.17	42.25	
		(6.82)	(6.89)	(6.95)	(5.74)	
	cocaine	37.18	39.94	43.75	32.04	
		(9.74)	(10.55)	(7.21)	(8.63)	
DEQ- high	control	10.77	12.17	7.17	7.17	
		(5.23)	(5.59)	(4.16)	(3.95)	
	cocaine	14.35	16.14	15.87	14.64	
		(5.45)	(5.67)	(5.78)	(5.44)	
DEQ- like more	control	25.77	25.62	32.92	25.65	
		(7.20)	(6.54)	(7.01)	(6.08)	
	cocaine	25.96	25.68	28.71	26.16	
		(9.18)	(8.99)	(8.48)	(8.69)	

Means (delta-method robust standard errors) for subjective effects measures (ARCI, DEQ, VAS) across placebo, 150 mg caffeine, 300 mg caffeine, and 20 mg d-amphetamine doses. Data are averaged across two time points on each dose day, and shown separately for control and cocaine-dependent groups

* *p* < .05, dose × time interaction, see text for details

** *p* < .05, dose × group interaction, see text for details

*** *p* < .05, dose × group × time interaction, see text for details

**Table 3:** Means (delta-method robust standard errors) for accuracy (selection of the stimulus with 80% “correct” feedback) during stage 1 training

Variable	Group	Dose			
		placebo	caffeine 150	caffeine 300	d-amph 20
accuracy *	control	0.56	0.55	0.56	0.61
		(0.02)	(0.03)	(0.02)	(0.02)
	cocaine	0.54	0.56	0.58	0.56
		(0.01)	(0.02)	(0.02)	(0.02)

(acquisition phase) on the probabilistic feedback selection task across placebo, 150 mg caffeine, 300 mg caffeine, and 20 mg d-amphetamine doses. Data are were collected approximately 75 min after dosing, and are shown separately for the control and cocaine-dependent groups

* *p* < .05 main effect of dose, see text for details
